# Prevalence of pituitary disorders associated with traumatic brain injury: a systematic review

**DOI:** 10.1186/cc10906

**Published:** 2012-03-20

**Authors:** F Lauzier, O Lachance, B Senay, I Côté, P Archambault, F Lamontagne, A Boutin, L Moore, F Bernard, C Gagnon, D Cook, AF Turgeon

**Affiliations:** 1 CHA-Hôpital de l'Enfant-Jésus, Université Laval, Québec, Canada; 2 Université Laval, Québec, Canada; 3 Université de Sherbrooke, Canada; 4 Université de Montréal, Canada; 5 McMaster University, Hamilton, Canada

## Introduction

Pituitary disorders are an often-neglected consequence of traumatic brain injury (TBI). We systematically reviewed their prevalence in studies with low risk of bias including moderate/severe TBI patients.

## Methods

We searched EMBASE, MEDLINE, Scopus, Cochrane Central Register, BIOSIS, Trip Database, references of included studies and narrative reviews. We included cohort studies, cross-sectional studies and RCTs that tested the integrity of ≥1 pituitary axis in adult victims of TBI. Two investigators independently reviewed selected citations, extracted data and assessed the risk of bias. Studies including <10% of mild TBI victims were considered as involving mainly moderate/severe TBI patients. Prevalence is reported as weighted mean (lowest and highest prevalence) in three time-frames: acute (<1 month post TBI), mid (3 to 12 months) and long-term setting (>12 months). Studies were considered at low risk of bias if the authors defined inclusion/exclusion criteria, avoided voluntary sampling, and tested >90% of included patients with proper detailed diagnostic criteria. Studies testing all pituitary axes were considered as evaluating hypopituitarism, which was defined as the dysfunction of at least one axis.

## Results

Among 12,514 citations, we included 55 studies (4,648 patients). Patients suffered from mild (11.9%, *n *= 555), moderate (7.9%, *n *= 367) and severe (30.4%, *n *= 1,415) TBI, others being of unknown severity. Prevalences of pituitary axis dysfunction are reported in Table 1. Few studies considering mainly moderate/severe TBI patients were at low risk of bias.

**Table 1:** 

	Hypopituitarism	GH	ACTH	TSH	Gonadal	ADH
Acute phase						
All studies,	58.3% (32.3 to 76.1),	28.7% (8.8 to 77.2),	14.3% (0.7 to 45.7),	9.4% (0 to 40.6),	44.3% (7.7 to 91.7),	12.6% (0 to 27.2),
*n *(patients)	6 (513)	9 (784)	11 (958)	12 (837)	10 (827)	13 (1821)
Low risk of bias,	71.3% (52.9 to 76.1),	36.8% (8.8 to 77.2),	14.5% (0.7 to 23.6),	10.1% (1.6 to 32.6),	54.3% (23.5 to 80.0),	18.8% (0 to 27.2),
*n *(patients)	3 (216)	5 (389)	4 (385)	6 (523)	5 (337)	5 (739)
+ moderate/severe,	70.0% (52.9 to 74.3),	36.1% (8.8 to 77.2),	14.5% (0.7 to 23.6),	5.2% (1.6 to 14.7),	61.4% (23.5 to 80.0),	23.8% (0 to 27.2),
*n *(patients)	2 (170)	4 (321)	4 (385)	4 (406)	3 (220)	4 (303)
Mid-term						
All studies,	32.1% (8.9 to 56.4),	14.8% (6.3 to 25.0),	9.7% (0 to 50.0),	4.3% (0 to 22.2),	18.8% (0 to 66.7),	3.8% (0 to 14.0),
*n *(patients)	9 (608)	11 (643)	12 (669)	11 (629)	15 (792)	11 (691)
Low risk of bias,	-	12.1% (6.3 to 22.2),	16.7% (4.2 to 50.0),	6.1% (0 to 22.2),	25.2% (0 to 56.3),	11.2% (8.3 to 14.0),
*n *(patients)		5 (231)	4 (215)	5 (231)	5 (218)	2 (98)
+ moderate/severe,	-	12.1% (6.32 to 22.2),	16.7% (4.2 to 50.0),	6.1% (0 to 22.2),	25.2% (0 to 56.3),	8.3% (-),
*n *(patients)		5 (231)	4 (215)	5 (231)	5 (218)	1 (48)
Long-term						
All studies,	29.1% (0.9 to 73.3),	15.0% (0 to 51.8),	10.2% (0 to 64.4),	6.3% (0 to 31.8),	12.2% (0 to 50.0),	2.7% (0 to 18.2),
*n *(patients)	19 (1418)	27 (1966)	26 (1782)	25 (1698)	25 (1798)	17 (1108)
Low risk of bias,	31.1% (-),	16.6% (7.2 to 28.0),	6.8% (0 to 18.8),	8.2% (1.0 to 20.0),	12.9% (1.5 to 29.3),	5.0% (0 to 6.9),
*n *(patients)	1 (45)	8 (499)	6 (369)	10 (734)	9 (707)	3 (200)
+ moderate/severe,	31.1% (-),	15.7% (7.2 to 21.7),	7.3% (1.4 to 18.8),	8.6% (1.0 to 20.0),	12.7% (1.5 to 29.3),	6.7% (6.3 to 6.9),
*n *(patients)	1 (45)	5 (381)	4 (301)	7 (616)	6 (589)	2 (150)

## Conclusion

Pituitary disorders frequently arise after TBI, but prevalence remains uncertain due to low overall quality of available data. Factors other than methodological quality and TBI severity are likely to explain the observed wide prevalence ranges. The clinical significance of TBI-associated pituitary disorders also requires further rigorous evaluation.

